# Round the Hospitals

**Published:** 1921-02-12

**Authors:** 


### ROUND THE HOSPITALS.

THE news that the British Red Cross Society has struck a medal for presentation to its numerous war workers who have received no British decoration or medal is likely to be very popular. Among those entitled to receive it are all members of the Society or its Voluntary-Aid Detachments who, during the war, gave a minimum unpaid service at home or abroad of not less than a thousand hours—a very sufficient test of good-will. In the case of ambulance drivers and bearers, the minimum number of hours is five hundred. For air-raid duty, in the course of which great personal danger was in many cases incurred, there is no fixed minimum period of service, and the Red Cross County Presidents will nominate for the medal in their discretion. The medal, which is in gilt with a white ribbed-silk ribbon, is the only one ever issued by the British Red Cross Society. Engraved on the obverse is the well-known symbol, with the words "For war service 1914-1918," while the reverse bears the Red Cross motto, "*Inter arma caritas.*" Forms of application can be obtained from the Secretary, British Red Cross Society, 19 Berkeley Street, W. 1. Envelopes should be marked B.R.C.S. Medal.

**ROUND THE HOSPITALS**—(*continued*).

The invaluable and unremitting labours of the Committee which administers the Nation's Fund for Nurses are too little known to nurses at large, who possibly believe that the money was got with little effort, and is spent with less. Their eyes are likely to be opened very shortly, when they and the public will learn a good deal about the admirable methods adopted to meet the necessities of the women who have spent themselves for others. Pending the publication of the full particulars, which are on their way, we say no more on this subject, but that, so far from being a labour completed in one gigantic effort, it is a steady, continuous movement likely to be needed while this generation lasts and entitled to the most open-handed generosity and loyal co-operation of the entire profession.

---

A sum of £25,000 is being asked for to establish a Home in Glasgow for Retired Nurses. A similar home in Edinburgh, with accommodation for fifteen nurses, has proved a great success, and the sister city has come to realise its own need. Glasgow nurses have not been lacking in helping to provide funds for the Edinburgh home, and they are now concentrating on a grand effort to build up a capital fund for the establishment of a Glasgow home, which shall yield a sum sufficient to meet any deficiency in carrying it on. The sale of work held towards this object, on February 5, in the nurses' drawing-room of the Western Infirmary, Glasgow, was a great inauguration, and Miss Gregory Smith and the present and former nurses of that institution are to be warmly congratulated on the success of their efforts. No less a sum than £2,400 was realised from this sale of work on one afternoon. The industry and organisation which have been employed to achieve such a splendid result must indeed have been great, and they deserve that the citizens of Glasgow should unhesitatingly adopt the suggestion referred to by Mrs. McCowan, who presided at the sale. This was that those whose daughters had safely returned from their nursing work during the war should show their gratitude by providing some of the £25,000 required to make the scheme a success. We hope that the sum aimed at will be promptly provided, for Glasgow citizens can have no better object for their wealth.

---

Lord Knutsford has expressed his entire agreement with the College of Nursing scale of salaries for trained nurses, but he thinks £18 is too little to give a probationer in her first year. At the London they give £30, and he thinks this is not too high. To meet cost of clothes, boots, recreation, and holiday £30 is not too much, and it is natural that at the age of twenty to twenty-three women should wish to be self-supporting. No doubt some hospitals can attract the candidates they need on the lower scale, but at present prices it is hardly reasonable for the rank-and-file institutions to expect to do so. Lord Knutsford is a strong advocate for paying pensions to nurses after twenty years' service in the institution. It is a wise and just policy, but it is not likely to make any impression on candidates. Few indeed of those who commence their training have any intention of remaining in the institution twenty years, nor, even if they wish, is it possible for any but a very small minority to do so?

---

The Infirmary Committee of the Camberwell Board of Guardians have had an application from the nursing staff asking for a reduction in their hours of duty. The matron, Miss M. E. Jones, in a report states that at present the day staff are working fifty-seven and a-half hours a week and the night staff seventy-two hours, each nurse getting one day and one night off duty. In a scheme she submitted the hours of the day nurses would be reduced to forty-eight and a-half a week and those of the night nurses to fifty-eight and three-quarters. This would necessitate the appointment of an extra night superintendent, twenty-seven probationers, two dormitory maids, two mess-room maids, and a kitchen maid and two laundry helpers, in addition to two more sisters whom the Guardians have already received sanction from the Ministry of Health to appoint. Extra accommodation would be required for the additional officers mentioned, and, as the committee have not completed their inquiries as to how this could best be procured, the guardians have decided to take no action until the complete scheme is presented to them.

---

Cavendish Square has become the centre of nursing interests in London. With the Cowdray Club at No. 20, the new United Nursing Services Club for members of the Naval and Military Nurses Services, and the British Red Cross Club for the V.A.D.s, the wide cross-roads will soon be dotted with varied uniforms, and, we trust, the grassy shades of the square enlivened by tennis players. The International character of the Cowdray Club should prove of real value to nursing interests all over the Empire, and, indeed, far beyond. That its motto will be reunion instead of exclusion is a most hopeful sign for the new era now dawning. It is such a meeting-ground as has hitherto been entirely lacking and greatly missed. Strangers to our shores cannot meet with the cordial reception we should all like them to get unless this mingled duty and pleasure be well organised by the right people, with command of suitable premises. A strong Hospitality Committee, or whatever it may be called, affiliated to nursing organisations in different quarters of the globe, is indicated.

---

Only recently the announcement was made that the medical superintendent of the Christchurch Hospital, New Zealand, has offered to found a scholarship for nurses trained in that institution who desire to take up the work of a sister-tutor. Another example of the progressive spirit in the Dominions is to hand this week. The Johannesburg Hospital, South Africa, as our advertisement

**ROUND THE HOSPITALS**—*(continued)*.

columns tell, desires a sister-tutor qualified at King's College for Women. Presumably its intention last autumn to send a Transvaal trained nurse to London to take the sister-tutor's course at King's College has not materialised. The candidate in the present instance will be required to sign an agreement for three years and railway fare and passage will be provided. Further particulars will be supplied by the Secretary of the College of Nursing. The hospital of which Miss Alexander is matron contains 667 beds, and in 1920 had a staff of two branch matrons, two assistant matrons, thirty-one sisters, fifty-six staff nurses, and 268 probationers. Here is a great opportunity for a woman who wishes to do useful work in and for her profession and at the same time to broaden her outlook and enrich her mind by a knowledge of life in the Dominions overseas.

---

The Queen Victoria Jubilee Institute for Nurses depends largely on the supplementary efforts of the Queen's Fund, which was started on rather unique lines to feed the parent society. The Duke of Portland took the chair at the annual meeting of this Fund on February 1 and described the methods adopted to raise the sum of £7,000 they set themselves to get two years ago. The Nation's Fund for Nurses has contributed £2,500; the Victory Ball furnished £2,000; the Joint Committee of the British Red Cross and the Order of St. John have contributed £5,000; while Queen Alexandra's Committee has contributed £2,000. The serious financial condition of the Jubilee Institute renders it necessary to raise £12,000 as soon as possible. The outlook, in fact, is grave.

---

An interesting function which comes round every year preceded the last meeting of the Board of Management of the Cardiff King Edward VII. Hospital, when the proficiency medals of gold, silver, and bronze respectively, provided by that good friend of the hospital, Sir William James Thomas, were ceremoniallly handed to the three nurses adjudged the most efficient during the preceding twelve months. The duty was discharged by Lady Diamond, wife of the Chairman of the Managing Board (in the absence of Lady Thomas through indisposition), who congratulated the successful nurses. On behalf of the Nursing Committee, of which he is Chairman, Dr. Ewen Maclean expressed a deep sense of the hospital's indebtedness to the whole body of nurses for the excellent work they had accomplished during the past year, and added that the winner of the gold medal was a thoroughly satisfactory all-round working nurse, whose work in the wards very clearly established, with other things, her claim to the outstanding position of gold medallist for the year. Dr. Maclean also paid a well-deserved tribute to the distinguished services of the matron, Miss E. Mont-Wilson.

---

The new maternity hospital at Southport is a notable addition to this progressive town. It has been fitted up in the pavilion, which during the war was used for the wounded, and as regards all nursing arrangements is under the control of the matron of the infirmary, and with a staff of fully-trained nurses and midwives. As a midwifery training centre it will be a useful addition to the infirmary, but financially it is quite independent of the Poor Law, the municipality defraying half the cost and the Ministry of Health the rest. Several of the speakers, both lay and medical, testified to the need for lying-in accommodation which it is hoped to supplement by the addition of a babies' observation ward. A charge of about £5 a case will be made, to be reduced or remitted in case of necessity.

---

Consideration has been given by the Maternity Committee of the Hammersmith Borough Council as to the best means of reducing as far as possible expenses at the maternity home. In this connection the Committee has ascertained that in many similar institutions fees are charged for training pupil midwives. The present arrangement at the Hammersmith Maternity Home provides for free training for a period of four months, and in consideration of this training the midwife, after passing the examination of the C.M.B., gives a further period of two months without salary. The Committee is of opinion that the present arrangement should be discontinued, and that a charge of twenty guineas should in future be made for the training received, the Council, out of this sum, to pay the pupil's necessary examination fees of five guineas. The Committee further suggests that the period of service should be reduced from six to four months, and adds that if this scheme is adopted a considerable income will be assured to the Council, which could be allocated to the expenses of the home of maternity work generally.

---

Princess Alice and the Earl of Athlone lent their aid recently to the Royal Waterloo Hospital for Women and Children by attending an At Home at the hospital in the absence of the Duchess of Albany, who has its interests warmly at heart. One of the most pressing needs is a Nurses' Home, but this is only part of a large extension scheme estimated to cost £80,000. The deficit on the working of the hospital amounts to £4,500, so there is need for all its friends to make a prolonged and energetic effort if all hopes are to be realised.

---

A strange state of things has come about at the Welshpool Hospital, where the matron and nurses have resigned and the hospital has in consequence been closed. Miss Trust, the matron, stated she was overworked and that the Ladies' House Committee had interfered with her in the discharge of her duties, expecting her to be matron, night nurse, day nurse and general servant. The Earl of Powis, taking the chair at the annual general meeting of subscribers, stated that as they were changing the staff the hospital was to be closed for structural repairs. There was evidence of considerable friction between the committee and the doctors.

